# Triglyceride-glucose index and the risk of stroke and its subtypes in the general population: an 11-year follow-up

**DOI:** 10.1186/s12933-021-01238-1

**Published:** 2021-02-18

**Authors:** Anxin Wang, Guangyao Wang, Qian Liu, Yingting Zuo, Shuohua Chen, Boni Tao, Xue Tian, Penglian Wang, Xia Meng, Shouling Wu, Yongjun Wang, Yilong Wang

**Affiliations:** 1 Department of Neurology, Beijing Tiantan Hospital, Capital Medical University, No.119, South 4th Ring West Road, Fengtai District, Beijing, 100070 China; 2 China National Clinical Research Center for Neurological Diseases, Beijing Tiantan Hospital, Capital Medical University, Beijing, China; 3 Graduate School, North China University of Science and Technology, Tangshan, China; 4 Department of Epidemiology and Health Statistics, School of Public Health, Capital Medical University, Beijing, China; 5 Department of Cardiology, Kailuan General Hospital, North China University of Science and Technology, No.57 Xinhua East Street, Lubei District, Tangshan, 063000 China; 6 School of Public Health, North China University of Science and Technology, Tangshan, China

**Keywords:** Insulin resistance, Triglyceride-glucose index, Stroke

## Abstract

**Background:**

Triglyceride-glucose (TyG) index was recently suggested to be a reliable surrogate marker of insulin resistance. We aim to investigate the associations between baseline and long-term TyG index with subsequent stroke and its subtypes in a community-based cohort.

**Methods:**

A total of 97,653 participants free of history of stroke in the Kailuan Study were included. TyG index was calculated as ln (fasting triglyceride [mg/dL] × fasting glucose [mg/dL]/2). Baseline TyG index was measured during 2006–2007. Updated cumulative average TyG index used all available TyG index from baseline to the outcome events of interest or the end of follow up. The outcome was the first occurrence of stroke, including ischemic stroke, intracerebral hemorrhage and subarachnoid hemorrhage. The associations of TyG index with outcomes were explored with Cox regression.

**Results:**

During a median of 11.02 years of follow-up, 5122 participants developed stroke of whom 4277 were ischemic stroke, 880 intracerebral hemorrhage, and 144 subarachnoid hemorrhage. After adjusting for confounding variables, compared with participants in the lowest quartile of baseline TyG index, those in the third and fourth quartile were associated with an increased risk of stroke (adjusted hazard ratio [HR] 1.22, 95% confidence interval [CI] 1.12–1.33, and adjusted HR 1.32, 95% CI 1.21–1.44, respectively, *P* for trend < 0.001). We also found a linear association between baseline TyG index with stroke. Similar results were found for ischemic stroke. However, no significant associations were observed between baseline TyG index and risk of intracranial hemorrhage. Parallel results were observed for the associations of updated cumulative average TyG index with outcomes.

**Conclusions:**

Elevated levels of both baseline and long-term updated cumulative average TyG index can independently predict stroke and ischemic stroke but not intracerebral hemorrhage in the general population during an 11-year follow-up.

## Background

Insulin resistance is a hallmark of metabolic syndrome and is considered to be one of the important risk factors of stroke [1, 2]. A reliable surrogate marker of insulin resistance was suggested to be the triglyceride-glucose (TyG) index, which was calculated as ln (fasting triglyceride [mg/dL] × fasting glucose [mg/dL]/2) [3, 4]. Some studies showed an association between TyG index and incidence of cardiovascular diseases [5, 6]. Current data about associations between TyG index and stroke are limited. A cohort study showed that higher levels of TyG index were not associated with higher risk of cerebrovascular disease, but it indicated that participants with higher levels of TyG index tended to have higher risk of events [7]. Recently, a cross-sectional study showed that the elevated levels of TyG index was associated with a higher risk of ischemic stroke in a general population [8]. In addition, the relationships of TyG index with the risk of future stroke subtypes are uncertain. Therefore, we aim to investigate the associations between TyG index and the occurrence of stroke and its subtypes in the Kailuan Study. We further evaluated the associations between updated cumulative average TyG index and stroke and its subtypes in the Kailuan Study.

## Methods

### Study design and participants

The Kailuan Study is a prospective cohort study conducted in the Kailuan community in Tangshan City, China [9]. The detailed design of the Kailuan study have been described previously [10]. Briefly, from June 2006 to October 2007, a total of 101,510 participants aged from 18 to 98 years in the community were enrolled in the Kailuan study and underwent a comprehensive biennial health examination at the Kailuan General Hospital. To minimize the possible effect of reverse causality, participants with a history of stroke were excluded. In addition, we excluded participants without fasting triglyceride and fasting blood glucose at baseline.

This study was approved by the Ethics Committee of the Kailuan General Hospital and Beijing Tiantan Hospital. All of the participants provided written informed consent.

### Data collection and definitions

Baseline data on demographics and clinical characteristics, including age, sex, body mass index, alcohol use, smoking status, income, education, blood pressure, physical activity, history of disease, and medications were collected via questionnaires by trained interviewers. Educational level was categorized as illiteracy or primary school, middle school, and high school or above. The high-income level was defined as participants’ average monthly income > 800 Renminbi/month. Smoking and drinking status was classified as three levels: never, former and current. Active physical activity was defined as “> 4 times per week and > 20 min at a time’’. Body mass index was calculated as weight (kg)/height (m)^2^. Systolic blood pressure and diastolic blood pressure was the average of three measurements in the seated position using a mercury sphygmomanometer.

Venous blood samples were obtained from overnight fasting participants. Serum specimens were stored in the central laboratory of the Kailuan General Hospital. All the serum specimens were measured on the Hitachi 747 auto-analyzer (Hitachi, Tokyo, Japan). Fasting blood glucose was measured by the hexokinase/glucose-6-phosphate dehydrogenase method. Serum triglyceride, high-density lipoprotein cholesterol (HDL-C) and low-density lipoprotein cholesterol (LDL-C) were measured by the enzymatic colorimetric method. Plasma high-sensitivity C-reactive protein (hs-CRP) were measured by using high-sensitivity particle-enhanced immunonephelometry assay.

### Assessment of TyG index

TyG index was calculated as ln (fasting triglyceride [mg/dL] × fasting glucose [mg/dL]/2) [11]. Fasting triglyceride and fasting glucose concentration were biennially measured from 2006 to 2017. Baseline TyG index was calculated by using fasting triglyceride and fasting glucose measured during 2006–2007. To represent long-term TyG index patterns of participants, we calculated updated cumulative average TyG index using all available TyG index measurements from 2006 to the outcome events of interest or the end of follow-up [12].

### Outcomes and follow-up

The outcome was the first occurrence of stroke, including ischemic stroke, intracerebral hemorrhage and subarachnoid hemorrhage according to the World Health Organization criteria [13]. Adjudication of incident stroke in the Kailuan Study was described previously [9]. In brief, participants were followed up at biennial routine medical examinations until December 31, 2017. Participants were interviewed face-to-face by trained research coordinators. The outcome was additionally confirmed by checking discharge summaries from the 11 hospitals and medical records from medical insurance. For the participants without face-to-face follow-ups, outcome information was obtained directly by checking death certificates from provincial vital statistics offices, discharge summaries and medical records.

### Statistical analysis

TyG index was categorized into four groups by quartiles. Categorical variables were presented as proportions and continuous variables were presented as medians with interquartile ranges (IQRs). Difference between quartiles of TyG index were compared using χ^2^ test for categorical variables and Wilcoxon or Kruskal–Wallis test for continuous variables.

The associations of baseline and updated cumulative average TyG index with outcomes were explored with Cox proportional hazards models. In the first model, we adjusted for age and sex. The second model was adjusted for model 1 plus level of education, income, smoking status, alcohol abuse, physical activity, and body mass index. The third model was adjusted for model 2 plus systolic blood pressure, diastolic blood pressure, history of myocardial infarction, hypertension, diabetes mellitus, and dyslipidemia, HDL-C, LDL-C, hs-CRP, antidiabetic drugs, lipid-lowering drugs and antihypertensive drugs. The hazard ratios (HRs) and 95% confidence intervals (CIs) were reported. Trend tests were performed in the regression models after the median TyG index values of each quartile were entered into the model and treated as a continuous variable.

Time to the event in each group of TyG index at baseline illustrated using Kaplan–Meier curve. Additionally, restricted cubic splines were performed to examine the shape of the associations between baseline TyG index and outcomes with five knots (at the 5th, 25th, 50th, 50th, 75th and 95th percentiles). The reference point for baseline TyG index was the median of the reference group, and the HR was adjusted for all confounding variables. The potential linear relationships of baseline TyG index with outcomes were explored.

Stratified analyses about associations between baseline TyG index and outcomes were conducted between participants with a history of diabetes mellitus and without a history of diabetes mellitus.

A 2-sided value of *P* < 0.05 was considered statistically significant. All analyses were performed with SAS software version 9.4 (SAS Institute Inc, Cary, NC).

## Results

A total of 101,510 participants completed the baseline survey in the Kailuan Study. After excluding 2571 participants with a history of stroke, and 1286 participants without fasting triglyceride and fasting blood glucose at baseline, a total of 97,653 participants were eligible for inclusion in this study (Additional file 1: Fig. S1). Baseline characteristics for participants excluded and included are shown in Additional file 2: Table S1. The median (IQR) age of participants included in this analysis was 51.67 (43.53–58.97) years. The median (IQR) of baseline TyG index was 8.58 (8.18–9.05). Table 1 shows the baseline characteristics of patients according to baseline TyG index quartiles. Participants with higher quartiles of TyG index were more likely to be older, male, have a higher BMI and blood pressure, have a lower education level and income, have a higher proportion of current smokers and current drinkers, have a higher proportion of history of myocardial infarction, diabetes mellitus, hypertension and hypercholesterolemia, have a higher level of fasting plasma glucose, triglycerides, LDL-C, hs-CRP, have a lower level of HDL-C, and have a higher proportion of antidiabetic drugs, lipid-lowering drugs and antihypertensive drugs. Table 2 shows the baseline characteristics of patients according to the quartiles of updated cumulative average TyG index.

**Table 1:** Baseline characteristics according to quartiles of baseline TyG index

Variable	Total	TyG index				*P* value
		Quartile 1 (3.61–8.18)	Quartile 2 (8.18–8.57)	Quartile 3 (8.57–9.05)	Quartile 4 (9.05–12.50)	
Participants, n	97,653	24,412	24,414	24,415	24,412	
Age, years	51.67 (43.53–58.97)	50.62 (41.69–58.57)	51.68 (43.52–58.97)	52.19 (44.13–59.44)	52.05 (44.33–58.74)	< 0.001
Male, n (%)	77,748 (79.62)	18,013 (73.79)	19,350 (79.26)	19,839 (81.26)	20,546 (84.16)	< 0.001
High school or above, n (%)	19,064 (20.21)	5751 (24.50)	4556 (19.23)	4526 (19.21)	4231 (17.94)	< 0.001
Income > 800 Renminbi/month, n (%)	13,466 (14.29)	3641 (15.52)	3172 (13.40)	3315 (14.07)	3338 (14.16)	< 0.001
Body mass index, kg/m^2^	24.84 (22.60–27.22)	23.14 (21.11–25.32)	24.44 (22.39–26.64)	25.43 (23.41–27.66)	26.30 (24.22–28.41)	< 0.001
Systolic blood pressure, mm Hg	130.00 (119.30–140.70)	120.00 (110.00–135.00)	129.30 (116.70–140.00)	130.00 (120.00–143.30)	131.30 (120.00–150.00)	< 0.001
Diastolic blood pressure, mm Hg	80.00 (78.70–90.00)	80.00 (70.70–85.00)	80.00 (77.30–90.00)	80.70 (79.30–90.00)	85.00 (80.00–92.00)	< 0.001
Current smoker, n (%)	32,577 (34.26)	7876 (33.34)	7615 (31.94)	8160 (34.29)	8926 (37.47)	< 0.001
Current alcohol use, n (%)	35,613 (37.44)	8839 (37.40)	8309 (34.84)	8867 (37.25)	9598 (40.28)	< 0.001
Active physical activity, n (%)	85,899 (91.27)	21,392 (91.35)	21,682 (91.75)	21,366 (90.82)	21,459 (91.16)	0.004
Myocardial infarction, n (%)	1105 (1.13)	196 (0.80)	245 (1.00)	312 (1.28)	352 (1.44)	< 0.001
Diabetes Mellitus, n (%)	2860 (2.93)	175 (0.72)	311 (1.27)	625 (2.56)	1749 (7.17)	< 0.001
Hypertension, n (%)	11,450 (11.73)	1720 (7.05)	2379 (9.74)	3255 (13.33)	4096 (16.78)	< 0.001
Hypercholesterolemia, n (%)	5429 (5.56)	691 (2.83)	996 (4.08)	1519 (6.22)	2223 (9.11)	< 0.001
Fasting plasma glucose, mmol/L	5.11 (4.66–5.71)	4.78 (4.38–5.20)	5.01 (4.62–5.46)	5.24 (4.80–5.81)	5.66 (5.02–6.90)	< 0.001
Triglycerides, mmol/L	1.27 (0.89–1.93)	0.70 (0.58–0.82)	1.10 (0.98–1.22)	1.56 (1.36–1.79)	2.77 (2.18–3.90)	< 0.001
HDL-C, mmol/L	1.51 (1.28–1.77)	1.54 (1.30–1.80)	1.53 (1.31–1.77)	1.49 (1.27–1.74)	1.47 (1.25–1.75)	< 0.001
LDL-C, mmol/L	2.33 (1.82–2.83)	2.13 (1.60–2.72)	2.38 (1.92–2.82)	2.41 (1.97–2.90)	2.38 (1.82–2.88)	< 0.001
Hs-CRP, mg/L	0.80 (0.30–2.19)	0.60 (0.21–1.90)	0.72 (0.29–2.00)	0.88 (0.33–2.20)	1.03 (0.40–2.63)	< 0.001
TyG index	8.58 (8.18–9.05)	7.91 (7.70–8.06)	8.39 (8.29–8.48)	8.79 (8.68–8.91)	9.46 (9.23–9.82)	< 0.001
Antidiabetic drugs, n (%)	2185 (2.24)	131 (0.54)	224 (0.92)	478 (1.96)	1352 (5.54)	< 0.001
Lipid-lowering drugs, n (%)	796 (0.82)	93 (0.38)	158 (0.65)	187 (0.77)	358 (1.47)	< 0.001
Antihypertensive drugs, n (%)	9895 (10.13)	1484 (6.08)	2036 (8.34)	2840 (11.63)	3535 (14.48)	< 0.001

Data are given as median (interquartile range) unless otherwise indicated

*HDL-C* high-density lipoprotein cholesterol, *LDL-C* low-density lipoprotein cholesterol, *Hs-CRP* high-sensitive C-reactive protein, *TyG index* triglyceride-glucose

**Table 2:** Baseline characteristics according to quartiles of updated cumulative average TyG index

Variable	Total	TyG index				*P* value
		Quartile 1 (4.67–8.31)	Quartile 2 (8.31–8.65)	Quartile 3 (8.65–9.06)	Quartile 4 (9.06–12.20)	
Participants, n	97,653	24,413	24,413	24,414	24,413	
Age, years	51.67 (43.53–58.97)	51.61 (42.65–60.31)	51.79 (43.61–59.21)	52.03 (44.05–58.95)	51.27 (43.63–57.64)	< 0.001
Male, n (%)	77,748 (79.62)	18,539 (75.94)	19,345 (79.24)	19,642 (80.45)	20,222 (82.83)	< 0.001
High school or above, n (%)	19,064 (20.21)	5375 (22.75)	4559 (19.29)	4626 (19.66)	4504 (19.15)	< 0.001
Income > 800 Renminbi/month, n (%)	13,466 (14.29)	3481 (14.74)	3214 (13.61)	3374 (14.35)	3397 (14.44)	0.004
Body mass index, kg/m^2^	24.84 (22.60–27.22)	23.04 (21.05–25.26)	24.39 (22.41–26.61)	25.45 (23.43–27.64)	26.37 (24.34–28.51)	< 0.001
Systolic blood pressure, mm Hg	130.00 (119.30–140.70)	120.70 (110.00–138.70)	129.00 (116.70–140.00)	130.00 (120.00–144.00)	130.00 (120.00–150.00)	< 0.001
Diastolic blood pressure, mm Hg	80.00 (78.70–90.00)	80.00 (70.70–86.00)	80.00 (76.70–90.00)	80.70 (79.30–90.00)	83.30 (80.00–91.30)	< 0.001
Current smoker, n (%)	32,577 (34.26)	7628 (32.10)	7667 (32.23)	8240 (34.72)	9042 (37.99)	< 0.001
Current alcohol use, n (%)	35,613 (37.44)	8539 (35.91)	8288 (34.83)	9038 (38.08)	9748 (40.95)	< 0.001
Active physical activity, n (%)	85,899 (91.27)	21,705 (92.04)	21,528 (91.32)	21,325 (90.85)	21,341 (90.87)	< 0.001
Myocardial infarction, n (%)	1105 (1.13)	214 (0.88)	276 (1.13)	289 (1.18)	326 (1.34)	< 0.001
Diabetes Mellitus, n (%)	2860 (2.93)	139 (0.57)	329 (1.35)	614 (2.52)	1778 (7.28)	< 0.001
Hypertension, n (%)	11,450 (11.73)	1700 (6.96)	2419 (9.91)	3224 (13.21)	4107 (16.82)	< 0.001
Hypercholesterolemia, n (%)	5429 (5.56)	631 (2.59)	1041 (4.26)	1440 (5.90)	2317 (9.49)	< 0.001
Fasting plasma glucose, mmol/L	5.11 (4.66–5.71)	4.84 (4.45–5.26)	5.03 (4.61–5.50)	5.20 (4.73–5.80)	5.60 (5.00–6.70)	< 0.001
Triglycerides, mmol/L	1.27 (0.89–1.93)	0.76 (0.60–0.96)	1.13 (0.92–1.38)	1.51 (1.19–1.93)	2.43 (1.75–3.56)	< 0.001
HDL-C, mmol/L	1.51 (1.28–1.77)	1.55 (1.31–1.81)	1.53 (1.30–1.78)	1.49 (1.27–1.74)	1.45 (1.24–1.72)	< 0.001
LDL-C, mmol/L	2.33 (1.82–2.83)	2.15 (1.63–2.71)	2.36 (1.90–2.81)	2.40 (1.96–2.90)	2.39 (1.85–2.90)	< 0.001
Hs-CRP, mg/L	0.80 (0.30–2.19)	0.59 (0.20–1.70)	0.72 (0.29–2.00)	0.89 (0.33–2.25)	1.10 (0.45–2.72)	< 0.001
TyG index	8.58 (8.18–9.05)	8.00 (7.74–8.23)	8.43 (8.22–8.62)	8.77 (8.53–9.01)	9.35 (9.03–9.76)	< 0.001
Antidiabetic drugs, n (%)	2185 (2.24)	97 (0.40)	229 (0.94)	488 (2.00)	1371 (5.62)	< 0.001
Lipid-lowering drugs, n (%)	796 (0.82)	81 (0.33)	148 (0.61)	204 (0.84)	363 (1.49)	< 0.001
Antihypertensive drugs, n (%)	9895 (10.13)	1446 (5.92)	2094 (8.58)	2809 (11.51)	3546 (14.53)	< 0.001

Data are given as median (interquartile range) unless otherwise indicated

*HDL-C* high-density lipoprotein cholesterol, *LDL-C* low-density lipoprotein cholesterol, *Hs-CRP* high-sensitive C-reactive protein, *TyG * triglyceride-glucose

**Fig. 1:**
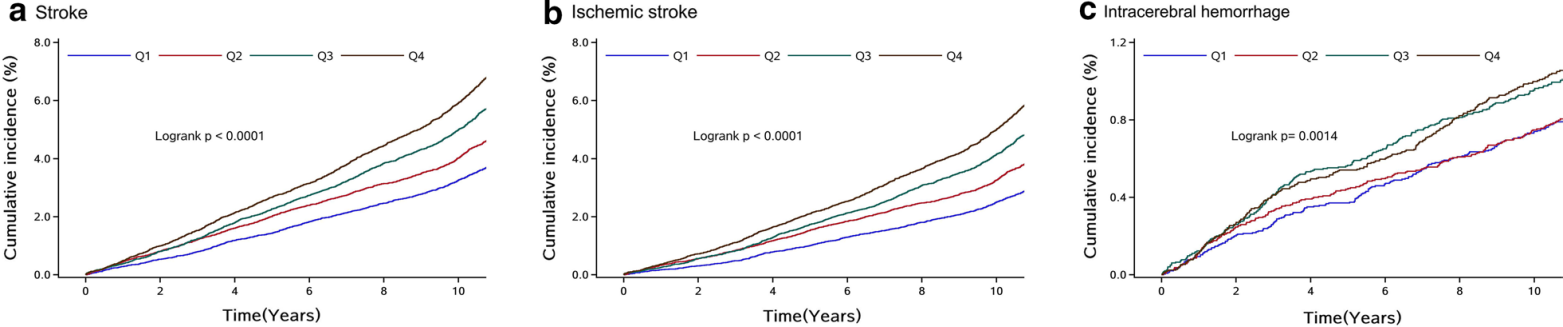
Kaplan–Meier curves of incidence of outcomes according to quartiles of baseline TyG index. **a** Stroke; **b** Ischemic stroke; **c** Intracerebral hemorrhage. Q1: quartile 1; Q2: quartile 2; Q3: quartile 3; Q4: quartile 4

**Fig. 2:**
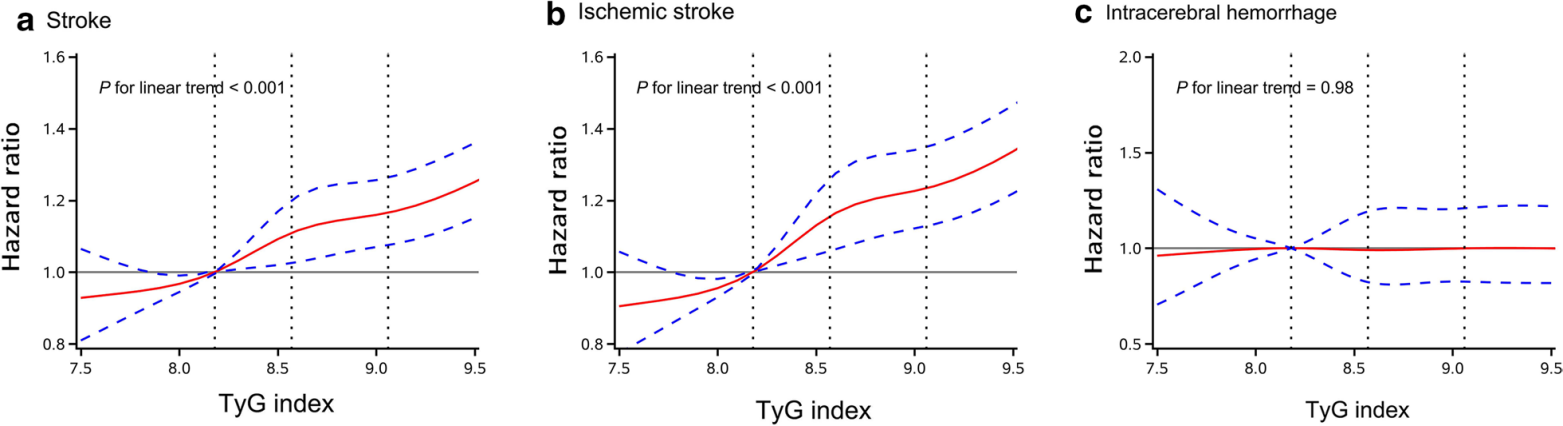
Adjusted hazard ratios of outcomes according to baseline TyG index. **a** Stroke; **b** Ischemic stroke; **c** Intracerebral hemorrhage. Data were fitted using a Cox regression model of restricted cubic spline with five knots (at the 5th, 25th, 50th, 50th, 75th and 95th percentiles) adjusting for potential covariates. The reference point for TyG index was the median of the reference group. Red lines indicate adjusted hazard ratio, and blue lines indicate the 95% confidence interval bands

After a median follow-up of 11.02 years (IQR 10.68–11.20 years), 5122 (5.25%) participants developed stroke, of whom 4277 were ischemic stroke, 880 intracerebral hemorrhage, and 144 subarachnoid hemorrhage. The incidence of stroke, ischemic stroke, intracerebral hemorrhage and subarachnoid hemorrhage was 5.05, 4.20, 0.85 and 0.14 per 1000 person-years, respectively. Participants with higher levels of baseline TyG index had a higher risk of stroke, ischemic stroke, and intracerebral hemorrhage (Fig. 1) (*P* < 0.05). After adjustment for potential confounding factors in the model 3, compared with participants with the lowest quartile of baseline TyG index, the adjusted HRs (95% CI) for stroke in the second, third, and highest quartiles of baseline TyG index were 1.08 (0.99–1.18), 1.22 (1.12–1.33), and 1.32 (1.21–1.44), respectively. The adjusted HRs (95% CI) for ischemic stroke in the second, third, and highest quartiles of baseline TyG index were 1.14 (1.03–1.26), 1.31 (1.19–1.44), and 1.45 (1.31–1.59), respectively. However, there were no significant associations between baseline TyG index and intracerebral hemorrhage, after adjustment for potential confounding factors in the model 3 (Table 3). Multivariable-adjusted spline regression models showed linear associations between baseline TyG index levels and the risk of stroke and ischemic stroke (Fig. 2). Similar results were observed for the association of updated cumulative average TyG index with outcomes (Table 4).

**Table 3:** HRs for risk of outcomes according to quartiles of baseline TyG index

Outcomes	TyG index				*P* value for trend
	Quartile 1 (3.61–8.18)	Quartile 2 (8.18–8.57)	Quartile 3 (8.57–9.05)	Quartile 4 (9.05–12.50)	
Stroke					
Case, n (%)	922 (3.78)	1139 (4.67)	1403 (5.75)	1658 (6.79)	
Incidence, per 1000 person-y	3.60	4.48	5.54	6.59	
Model 1	Reference	1.20 (1.10–1.31)	1.46 (1.35–1.59)	1.79 (1.65–1.93)	< 0.001
Model 2	Reference	1.14 (1.05–1.25)	1.34 (1.23–1.46)	1.58 (1.45–1.72)	< 0.001
Model 3	Reference	1.08 (0.99–1.18)	1.22 (1.12–1.33)	1.32 (1.21–1.44)	< 0.001
Ischemic stroke					
Case, n (%)	725 (2.97)	944 (3.87)	1183 (4.85)	1425 (5.84)	
Incidence, per 1000 person-y	2.82	3.70	4.65	5.64	
Model 1	Reference	1.26 (1.15–1.39)	1.57 (1.43–1.72)	1.96 (1.79–2.14)	< 0.001
Model 2	Reference	1.21 (1.10–1.33)	1.44 (1.31–1.58)	1.73 (1.58–1.90)	< 0.001
Model 3	Reference	1.14 (1.03–1.26)	1.31 (1.19–1.44)	1.45 (1.31–1.59)	< 0.001
Intracerebral hemorrhage					
Case, n (%)	190 (0.78)	193 (0.79)	244 (1.00)	253 (1.04)	
Incidence, per 1000 person-y	0.73	0.75	0.95	0.98	
Model 1	Reference	0.98 (0.80–1.20)	1.22 (1.01–1.48)	1.30 (1.07–1.56)	< 0.001
Model 2	Reference	0.93 (0.76–1.14)	1.13 (0.93–1.37)	1.16 (0.95–1.41)	0.04
Model 3	Reference	0.89 (0.73–1.09)	1.03 (0.85–1.25)	0.97 (0.79–1.18)	0.84

Model 1, adjusted for age and sex

Model 2, adjusted for variables in model 1 plus level of education, income, smoking, alcohol abuse, physical activity, and body mass index

Model 3, adjusted for variables in model 2 plus systolic blood pressure, diastolic blood pressure, history of myocardial infarction, hypertension, diabetes mellitus, and dyslipidemia, high-density lipoprotein cholesterol, low-density lipoprotein cholesterol, high-sensitive C-reactive protein, antidiabetic drugs, lipid-lowering drugs and antihypertensive drugs

TyG indicates triglyceride-glucose

**Table 4:** HRs for risk of outcomes according to quartiles of updated cumulative average TyG index

Outcomes	TyG index				*P* value for trend
	Quartile 1 (4.67–8.31)	Quartile 2 (8.31–8.65)	Quartile 3 (8.65–9.06)	Quartile 4 (9.06–12.20)	
Stroke					
Case, n (%)	1056 (4.33)	1157 (4.74)	1291 (5.29)	1618 (6.63)	
Incidence, per 1000 person-y	4.18	4.54	5.06	6.42	
Model 1	Reference	1.12 (1.03–1.22)	1.27 (1.17–1.37)	1.71 (1.58–1.85)	< 0.001
Model 2	Reference	1.06 (0.98–1.16)	1.16 (1.06–1.26)	1.50 (1.38–1.63)	< 0.001
Model 3	Reference	1.01 (0.93–1.10)	1.05 (0.96–1.14)	1.25 (1.15–1.36)	< 0.001
Ischemic stroke					
Case, n (%)	830 (3.40)	963 (3.94)	1089 (4.46)	1395 (5.71)	
Incidence, per 1000 person-y	3.27	3.76	4.25	5.51	
Model 1	Reference	1.19 (1.08–1.31)	1.37 (1.25–1.50)	1.89 (1.73–2.06)	< 0.001
Model 2	Reference	1.13 (1.03–1.24)	1.25 (1.14–1.37)	1.66 (1.51–1.81)	< 0.001
Model 3	Reference	1.07 (0.98–1.18)	1.13 (1.03–1.24)	1.39 (1.27–1.52)	< 0.001
Intracerebral hemorrhage					
Case, n (%)	219 (0.90)	203 (0.83)	214 (0.88)	244 (1.00)	
Incidence, per 1000 person-y	0.85	0.78	0.83	0.95	
Model 1	Reference	0.94 (0.78–1.14)	1.00 (0.83–1.21)	1.20 (1.00–1.44)	0.04
Model 2	Reference	0.89 (0.73–1.08)	0.92 (0.76–1.11)	1.06 (0.88–1.29)	0.45
Model 3	Reference	0.85 (0.70–1.03)	0.83 (0.68–1.00)	0.89 (0.73–1.08)	0.26

Model 1, adjusted for age and sex

Model 2, adjusted for variables in model 1 plus level of education, income, smoking, alcohol abuse, physical activity, and body mass index

Model 3, adjusted for variables in model 2 plus systolic blood pressure, diastolic blood pressure, a history of myocardial infarction, hypertension, diabetes mellitus, and dyslipidemia, high-density lipoprotein cholesterol, low-density lipoprotein cholesterol, high-sensitive C-reactive protein, antidiabetic drugs, lipid-lowering drugs and antihypertensive drugs. TyG indicates triglyceride-glucose

In our secondary analysis, Cox regression models were recalculated in participants with and without a history of diabetes mellitus (Additional file 2: Table S2). Higher quartiles of baseline TyG index were associated with an increased risk of stroke and ischemic stroke in participants without a history of diabetes mellitus. Baseline TyG index were not associated with outcomes in participants with a history of diabetes mellitus. There was no interaction between baseline TyG index and with or without a history of diabetes mellitus for the risk of outcomes (*P* for interaction > 0.05).

## Discussion

In this large, prospective, population-based cohort study, we found that higher levels of TyG index at baseline were associated with an increased risk of future stroke and ischemic stroke during an 11-year follow-up. However, there was no significant association between baseline TyG index and intracerebral hemorrhage. Similar results were observed for the associations of long-term updated cumulative average TyG index with outcomes.

Former studies indicated that TyG index was associated with cardiovascular disease. A retrospective cohort study showed that elevated levels of TyG index were associated with an increased risk of cardiovascular disease in participants aged over 60 years [6]. Higher levels of TyG index were significantly associated with an increased risk of cardiovascular events and coronary heart disease during long-term follow‑up [5]. TyG index was also an independent predictor of coronary artery calcification progression [11], coronary artery disease severity and cardiovascular outcomes [14]. However, data about associations between TyG index and stroke and its subtypes are limited. A cohort study with 5014 apparently healthy participants indicated that higher levels of TyG index were not significantly associated with cerebrovascular disease, but it indicated that those with higher levels of TyG index tended to have higher risk of cerebrovascular disease [7]. In this current study, the incidence of stroke increased according to TyG index levels. We also found that the both baseline and long-term updated cumulative average TyG index were independently associated the risk of stroke and ischemic stroke. This lack of statistical significance in that previous cohort study may be explained by the smaller number of outcome events.

The mechanisms accounting for the associations between TyG index and stroke and ischemic stroke remain unclear. TyG index is a surrogate marker of insulin resistance, which may mainly account for these associations [1, 15–17]. Firstly, insulin resistance led to the chronic inflammation [18], endothelium dysfunction [19–21], facilitated the formation of foam cells in the initiation of atherosclerosis [22] and promoted the vulnerable plaque [23]. Previous studies indicated that TyG index was associated with increased arterial stiffness in general population [24, 25] and an independent predictive marker for plaque progress [26], which could contribute to the occurrence of stroke. Secondly, previous research showed that insulin resistance affected platelet adhesion, activation and aggregation [27–29] which were associated with artery stenosis or occlusion and involved in stroke occurrence. Higher TyG index levels were associated with an increased risk of coronary artery stenosis and the number and the severity of artery stenoses [30, 31]. Thirdly, participants with higher quartiles of TyG index were more likely to be older, male, have a higher BMI, have a higher proportion of current smokers and history of myocardial infarction, diabetes mellitus, hypertension and hypercholesterolemia, have a higher level of fasting plasma glucose, triglycerides, LDL-C and hs-CRP, which were a cluster of risk factors for stroke [32–37]. Insulin resistance could modify and influence the role of the stroke risk factors and contribute to stroke [17].

Participants with higher levels of baseline and updated cumulative average TyG index had a higher risk of intracerebral hemorrhage, after adjusted for age and sex. However, statistical relevance was not significant for intracerebral hemorrhage in the multivariate adjusted models. Previous studies showed that insulin resistance measured by HOMA-IR [38, 39] and euglycaemic insulin clamp [40] was not associated with intracerebral hemorrhage, which was consist with our results. Hypertension, intake of antithrombotic drugs and amyloid angiopathy were the most important risk factors for intracerebral hemorrhage [41], which may explain for this nonsignificant association.

There are some limitations in this study. First, fasting insulin was not measured in the Kailuan Study and HOMA-IR could not be evaluated. Therefore, we could not compare the predictive value of TyG index and HOMA-IR for occurrence of stroke. Second, participants at higher levels of TyG index with a cluster of vascular risk factors were more likely to take low fat diet, take more exercise, control blood pressure and weight, quit smoking and restrict the intake of alcohol, according to the medical advice. Third, the study was not nationally representative. All participants were recruited from northern China and most of them were men.

## Conclusions

Elevated levels of both baseline and long-term updated cumulative average TyG index were independent predictors for stroke and ischemic stroke instead of intracerebral hemorrhage in the general population during an 11-year follow-up.

## Supplementary Information

**Additional file 1: Fig. S1.** Flow chart of the present study.

**Additional file 2: Table S1.** Baseline characteristics for participants excluded and included. **Table S2.** HR for risk of outcomes according to quartiles of baseline TyG index stratified by history of diabetes mellitus status.

## Data Availability

The datasets during and/or analysed during the current study available from the corresponding author on reasonable request.

## Abbreviations

TyG: Triglyceride-glucose
HDL-C: High-density lipoprotein cholesterol
LDL-C: Low-density lipoprotein cholesterol
Hs-CRP: High-sensitivity C-reactive protein
HR: Hazard ratio
CI: Confidence interval
IQR: Interquartile range
