# Supplementary material for: Triglyceride-glucose index and the risk of stroke and its subtypes in the general population: an 11-year follow-up
Source: Cardiovasc Diabetol. 2021 Feb 18;20:46. doi: 10.1186/s12933-021-01238-1 (PMC7893902; doi:10.1186/s12933-021-01238-1)
Supplement: Supplementary file 1 — Additional file 1: Fig. S1. Flow chart of the present study. [file 12933_2021_1238_MOESM1_ESM.pdf]

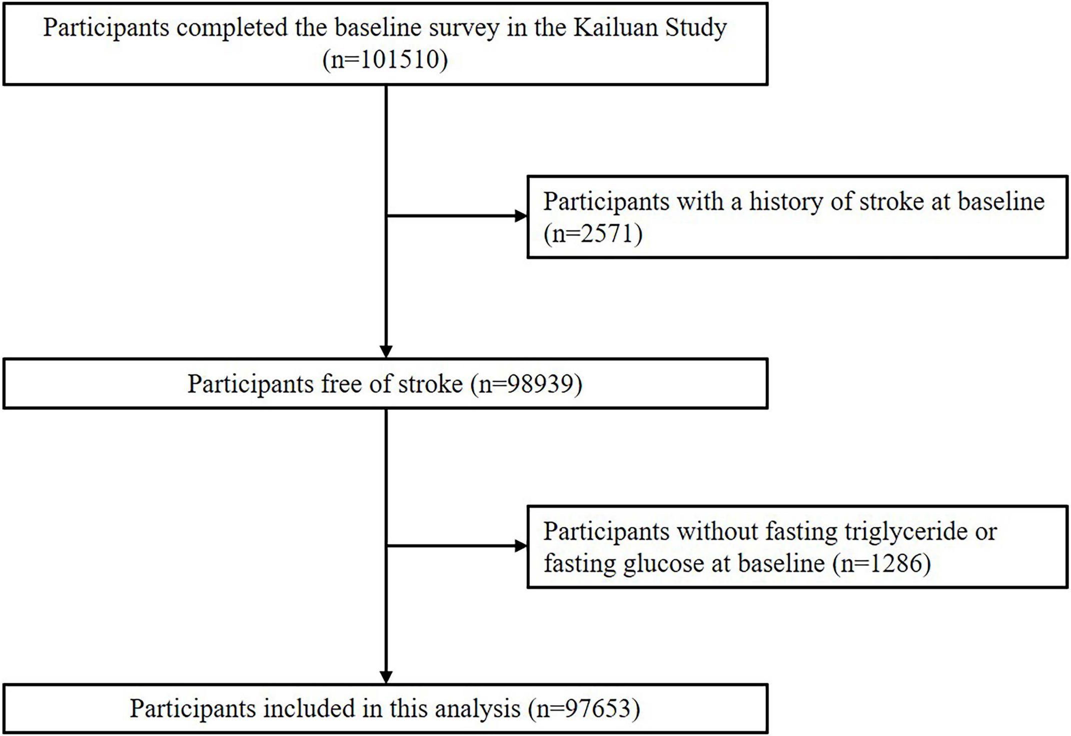
Participants completed the baseline survey in the Kailuan Study (n=101510)
Participants with a history of stroke at baseline (n=2571)
Participants free of stroke (n=98939)
Participants without fasting triglyceride or fasting glucose at baseline (n=1286)
Participants included in this analysis (n=97653)
