# Supplementary material for: Triglyceride-glucose index and the risk of stroke and its subtypes in the general population: an 11-year follow-up
Source: Cardiovasc Diabetol. 2021 Feb 18;20:46. doi: 10.1186/s12933-021-01238-1 (PMC7893902; doi:10.1186/s12933-021-01238-1)
Supplement: Supplementary file 2 — Additional file 2: Table S1. Baseline characteristics for participants excluded and included. Table S2. HR for risk of outcomes according to quartiles of baseline TyG index stratified by history of diabetes mellitus status. [file 12933_2021_1238_MOESM2_ESM.docx]

**Additional File**

**Additional tables**

**Table S1** Baseline characteristics for participants excluded and included

| Variable | All | Not included | Included | *P* value |
| --- | --- | --- | --- | --- |
| Participants, n | 101510 | 3857 | 97653 |  |
| Age, years | 51.95(43.69-59.42) | 60.73(54.02-68.78) | 51.67(43.53-58.97) | <0.001 |
| Male, n (%) | 81110(79.90) | 3362(87.17) | 77748(79.62) | <0.001 |
| High school or above, n (%) | 19611(20.11) | 547(17.06) | 19064(20.21) | <0.001 |
| Income >800 Renminbi/month, n (%) | 14015(14.38) | 549(17.14) | 13466(14.29) | <0.001 |
| Body mass index, kg/m2 | 24.86(22.64-27.22) | 25.39(23.05-27.68) | 24.84(22.60-27.22) | <0.001 |
| Systolic blood pressure, mm Hg | 130.00(119.30-141.30) | 140.00(123.30-159.30) | 130.00(119.30-140.70) | <0.001 |
| Diastolic blood pressure, mm Hg | 80.00(78.70-90.00) | 84.85(79.30-94.00) | 80.00(78.70-90.00) | <0.001 |
| Current smoker, n (%) | 33795(34.38) | 1218(37.91) | 32577(34.26) | <0.001 |
| Current alcohol use, n (%) | 36652(37.28) | 1039(32.38) | 35613(37.44) | <0.001 |
| Active physical activity, n (%) | 88822(91.27) | 2923(91.14) | 85899(91.27) | 0.80 |
| Myocardial infarction, n (%) | 1316(1.30) | 211(5.47) | 1105(1.13) | <0.001 |
| Diabetes Mellitus, n (%) | 3250(3.20) | 390(10.11) | 2860(2.93) | <0.001 |
| Hypertension, n (%) | 13004(12.81) | 1554(40.29) | 11450(11.73) | <0.001 |
| Hypercholesterolemia, n (%) | 6260(6.17) | 831(21.55) | 5429(5.56) | <0.001 |
| Fasting plasma glucose, mmol/L | 5.11(4.66-5.72) | 5.20(4.56-6.08) | 5.11(4.66-5.71) | 0.003 |
| Triglycerides, mmol/L | 1.27(0.90-1.93) | 1.42(1.00-2.05) | 1.27(0.89-1.93) | <0.001 |
| HDL-C, mmol/L | 1.50(1.28-1.76) | 1.46(1.24-1.72) | 1.51(1.28-1.77) | <0.001 |
| LDL-C, mmol/L | 2.33(1.82-2.83) | 2.27(1.75-2.90) | 2.33(1.82-2.83) | 0.25 |
| Hs-CRP, mg/L | 0.80(0.30-2.20) | 1.40(0.59-3.35) | 0.80(0.30-2.19) | <0.001 |
| TyG index | 8.58(8.19-9.06) | 8.70(8.30-9.15) | 8.58(8.18-9.05) | <0.001 |
| Antidiabetic drugs, n (%) | 2481(2.44) | 296(7.68) | 2185(2.24) | <0.001 |
| Lipid-lowering drugs, n (%) | 963(0.95) | 167(4.33) | 796(0.82) | <0.001 |
| Antihypertensive drugs, n (%) | 11314(11.15) | 1419(36.80) | 9895(10.13) | <0.001 |

Abbreviations: HDL-C indicates high-density lipoprotein cholesterol; LDL-C, low-density lipoprotein cholesterol; hs-CRP, high-sensitive C-reactive protein; TyG, triglyceride-glucose.

Data are given as median (interquartile range) unless otherwise indicated.

**Table S2** HRs for risk of outcomes according to quartiles of baseline TyG index stratified by history of diabetes mellitus status

| Outcomes | TyG index | | | | *P* value for trend | *P* value for interaction |
| --- | --- | --- | --- | --- | --- | --- |
|  | Quartile 1  (3.61-8.18) | Quartile 2  (8.18-8.57) | Quartile 3  (8.57-9.05) | Quartile 4  (9.05-12.50) |  |  |
| With a history of diabetes mellitus |  |  |  |  |  |  |
| Stroke |  |  |  |  |  |  |
| Case, n (%) | 15(8.57) | 34(10.93) | 64(10.24) | 216(12.35) |  |  |
| Incidence, per 1000 person-y | 8.74 | 8.01 | 8.27 | 11.40 |  |  |
| Model 1 | Reference | 1.29(0.70-2.36) | 1.27(0.72-2.23) | 1.58(0.94-2.68) | 0.03 | 0.90 |
| Model 2 | Reference | 1.27(0.69-2.34) | 1.25(0.71-2.21) | 1.56(0.92-2.64) | 0.04 | 0.94 |
| Model 3 | Reference | 1.18(0.64-2.16) | 1.13(0.64-1.99) | 1.34(0.79-2.28) | 0.16 | 0.93 |
| Ischemic stroke |  |  |  |  |  |  |
| Case, n (%) | 13(7.43) | 33(10.61) | 57(9.12) | 191(10.92) |  |  |
| Incidence, per 1000 person-y | 7.53 | 10.90 | 9.36 | 11.40 |  |  |
| Model 1 | Reference | 1.45(0.76-2.76) | 1.32(0.72-2.40) | 1.64(0.93-2.87) | 0.06 | 0.69 |
| Model 2 | Reference | 1.44(0.76-2.75) | 1.31(0.72-2.41) | 1.62(0.92-2.86) | 0.07 | 0.77 |
| Model 3 | Reference | 1.33(0.70-2.53) | 1.18(0.64-2.17) | 1.38(0.78-2.44) | 0.27 | 0.81 |
| Intracerebral hemorrhage |  |  |  |  |  |  |
| Case, n (%) | 3(1.71) | 1(0.32) | 7(1.12) | 27(1.54) |  |  |
| Incidence, per 1000 person-y | 1.68 | 0.31 | 1.11 | 1.55 |  |  |
| Model 1 | Reference | 0.19(0.02-1.78) | 0.66(0.17-2.55) | 0.92(0.28-3.05) | 0.29 | 0.43 |
| Model 2 | Reference | 0.17(0.02-1.66) | 0.61(0.16-2.40) | 0.86(0.25-2.88) | 0.33 | 0.43 |
| Model 3 | Reference | 0.15(0.02-1.48) | 0.53(0.13-2.10) | 0.81(0.24-2.75) | 0.30 | 0.38 |
| Without a history of diabetes mellitus |  |  |  |  |  |  |
| Stroke |  |  |  |  |  |  |
| Case, n (%) | 907(3.74) | 1105(4.58) | 1339(5.63) | 1442(6.36) |  |  |
| Incidence, per 1000 person-y | 3.57 | 4.40 | 5.42 | 6.14 |  |  |
| Model 1 | Reference | 1.19(1.09-1.30) | 1.45(1.33-1.58) | 1.70(1.57-1.85) | <0.001 |  |
| Model 2 | Reference | 1.13(1.04-1.24) | 1.32(1.21-1.44) | 1.50(1.37-1.63) | <0.001 |  |
| Model 3 | Reference | 1.08(0.99-1.18) | 1.22(1.12-1.33) | 1.31(1.20-1.43) | <0.001 |  |
| Ischemic stroke |  |  |  |  |  |  |
| Case, n (%) | 712(2.94) | 911(3.78) | 1126(4.73) | 1234(5.44) |  |  |
| Incidence, per 1000 person-y | 2.79 | 3.61 | 4.53 | 5.23 |  |  |
| Model 1 | Reference | 1.25(1.13-1.38) | 1.56(1.42-1.71) | 1.86(1.70-2.04) | <0.001 |  |
| Model 2 | Reference | 1.19(1.08-1.32) | 1.42(1.29-1.56) | 1.63(1.48-1.80) | <0.001 |  |
| Model 3 | Reference | 1.14(1.03-1.25) | 1.31(1.19-1.45) | 1.44(1.31-1.59) | <0.001 |  |
| Intracerebral hemorrhage |  |  |  |  |  |  |
| Case, n (%) | 187(0.77) | 192(0.80) | 237(1.00) | 226(1.00) |  |  |
| Incidence, per 1000 person-y | 0.73 | 0.75 | 0.94 | 0.94 |  |  |
| Model 1 | Reference | 1.00(0.82-1.22) | 1.23(1.02-1.49) | 1.27(1.05-1.54) | 0.003 |  |
| Model 2 | Reference | 0.95(0.77-1.16) | 1.14(0.93-1.38) | 1.14(0.93-1.39) | 0.07 |  |
| Model 3 | Reference | 0.91(0.74-1.11) | 1.04(0.85-1.27) | 0.96(0.78-1.17) | 0.99 |  |

Model 1, adjusted for age and sex.

Model 2, adjusted for variables in model 1 plus level of education, income, smoking, alcohol abuse, physical activity, and body mass index.

Model 3, adjusted for variables in model 2 plus systolic blood pressure, diastolic blood pressure, history of myocardial infarction, hypertension, and dyslipidemia, high-density lipoprotein cholesterol, low-density lipoprotein cholesterol, high-sensitive C-reactive protein, antidiabetic drugs, lipid-lowering drugs and antihypertensive drugs.

Abbreviations: TyG indicates triglyceride-glucose.

**Additional Figures**

**Figure legends**

**Fig. S1** Flow chart of the present study.
